# How to exploit the recycling system of a cell

**DOI:** 10.7554/eLife.105995

**Published:** 2025-02-21

**Authors:** Sasha L Evans, Bethany A Haynes, Delia Capatina, Rivka L Isaacson

**Affiliations:** 1 Department of Chemistry, Faculty of Natural, Mathematical and Engineering Sciences, King’s College London London United Kingdom

**Keywords:** drug discovery, E3 ligases, APC/C, D-Box peptides, E3 inhibitors, Human

## Abstract

Nature has inspired the design of improved inhibitors for cancer-causing proteins.

**Related research article** Eapen R, Okoye C, Stubbs C, Schimpl M, Tischer T, McCall E, Zacharopoulou M, Ferrer F, Barford D, Spring D, Lindon C, Phillips C, Itzhaki LS. 2025. Development of D-box peptides to inhibit the Anaphase Promoting Complex/Cyclosome. eLife **14**:RP104238. doi: 10.7554/eLife.104238.

Each of our trillions of cells bustles with activity in a carefully choreographed quest to keep us healthy. At their core, quality control mechanisms ensure that the right proteins are in the right place at the right time and that they are rapidly removed and recycled when necessary (Thompson and De-Souza, 2023). This relies on waste disposal systems such as the ubiquitin-proteasome pathway. In a modern therapeutic twist, these systems can sometimes be hijacked to specifically remove disease-causing proteins (Koszła and Sołek, 2024).

Ubiquitin is a small molecule aptly named for its ubiquitous presence throughout the body. It serves as a molecular ‘rubbish tag’, with long chains of ubiquitin being added onto a protein to earmark it for destruction. This labelling process requires three types of enzymes working in sequence. E1s and E2s first work generically across the cell to activate and prepare ubiquitin for attachment. E3 ligases then ensure that the tag is added to the right protein; as such, hundreds of specific human E3 enzymes exist, each with precise targets that ensure specificity (Müller and Hoppe, 2024).

E3 ligases are considered important candidates for modern therapeutic development, as they are mutated or present in abnormal levels in a range of diseases. APC/C (short for anaphase promoting complex/cyclosome), for example, operates during cell division and is implicated in certain types of cancer (Greil et al., 2022). E3 inhibitors, created to bind the enzymes and stop them from acting on their targets, therefore represent a promising approach. However, designing compounds that can act on E3 ligases is often a challenge. The activity of these large, multi-unit enzymes heavily relies on flexible regions that lack the neat crevices into which small molecules can be designed to fit. Various strategies are afoot to try and get past these problems (Rodríguez-Gimeno and Galdeano, 2025). Now, in eLife, Laura Itzhaki and colleagues – including Rohan Eapen as first author – report important advances in designing compounds that can block E3 activity (Eapen et al., 2025b).

The researchers, who are based at the University of Cambridge, AstraZenecaCambridge, the MRC Laboratory of Molecular Biology and the p53 Laboratory in Singapore, focused their efforts on the enzyme APC/C and its substrates (Okoye et al., 2022; Höfler et al., 2024). They zoned in on Cdc20, a key region in APC/C that recognises a motif, aptly known as the Destruction box (D-Box), on proteins targeted for disposal (Okoye et al., 2022). Binding takes place by coordinating multiple such weak interactions, a common biological strategy that confers strength in numbers while allowing for versatility (Isaacson and Díaz-Moreno, 2018). The goal here was to create molecules that would bind tightly to Cdc20, displacing its natural substrate.

The team started by designing ‘D-Box peptides’ that could bind to this APC/C region, based on analyses of the protein regions it naturally recognises. Different methods were applied to measure how strongly each of these peptides could associate with APC/C. This, in turn, revealed which amino acids were most important for tight binding. Peptide design was refined by introducing unnatural amino acids thought to provide a better fit and stronger binding. These are distinct from the 20 amino acids naturally encoded by our genes and include additional chemical groups that can nestle more closely into the protein’s surface. The atomic structure of the best four candidates was examined via X-ray crystallography, revealing where and how the interactions occurred (Figure 1). Further experiments in live cells confirmed that the D-Box peptides could stabilise APC/C and inhibit its function.

**Figure 1.**
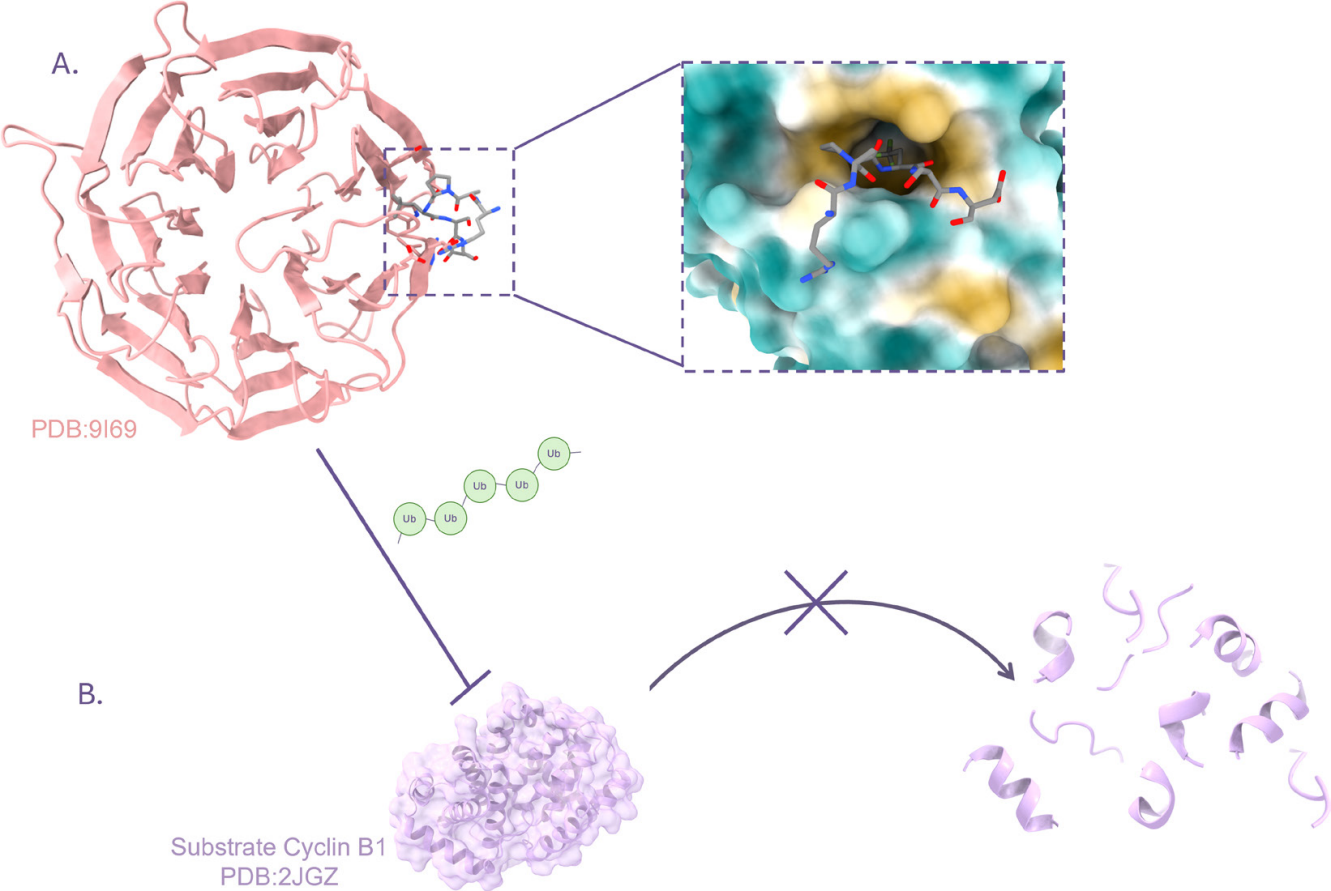
Designing artificial peptides that block the activity of the E3 ligase APC/C. (**A**) The E3 ligase APC/C is often implicated in cancers. It targets certain proteins important for cell division, such as Cyclin B1. Eapen et al. focused on a region in APC/C, known as the Cdc20 WD40 domain (pink banded structure), which is part of an element that recognises ‘D-box’ regions in target substrates. Based on information from natural APC/C, the team designed artificial peptides (inset), such as the D20 peptide (grey), that could bind to Cdc20 by recognizing a hydrophobic pocket (yellow) which is part of the Cdc20 D-box (whose hydrophilic areas are in turquoise). (**B**) The binding of the D20 peptide to the Cdc20 D-box prevents the addition of chains of ubiquitin (green) onto the APC/C substrate Cyclin B1 (lilac). Loss of ubiquitination prevents Cyclin B1 from being targeted for degradation.

Image credit: Crystal structure of human Cdc20 WD40 domain bound to synthetic D-box peptide D20 based on data deposited by Eapen et al., 2025a (CC BY 4.0); crystal structure of Cyclin B1 created using atomic coordinates deposited by Brown et al., 2007 (CC0). Figure created using Chimera X (Meng et al., 2023).

These findings represent an exciting launch pad for a range of scientific tools and potential therapeutics. Beyond the design of E3 inhibitors, these results could also inform the design of PROTACs, a new class of drugs launched in 2001 that feature a module designed to bind a specific target, such as a cancer-causing protein, attached to a module that recruits the ubiquitin-proteasome pathway to destroy it (Li and Crews, 2022). As for E3 inhibitors, the hope is that PROTACs have minimal side effects as they target only the disease culprit and nothing else. However, the challenge to find effective and selective versions of both modules persists. The work by Eapen et al. potentially offers solutions to the refinement of these promising therapeutics.

## References

[bib1] Brown NR, Lowe ED, Petri E, Skamnaki V, Antrobus R, Johnson LN (2007). Worldwide Protein Data Bank.

[bib2] Eapen R, Okoye C, Stubbs C, Schimpl M, Tischer T, Fisher EJ, Zacharopoulou M, Ferrer F, Barford D, Spring D, Lindon C, Phillips C, Itzhaki LS (2025a). Worldwide Protein Data Bank.

[bib3] Eapen R, Okoye C, Stubbs C, Schimpl M, Tischer T, McCall E, Zacharopoulou M, Ferrer F, Barford D, Spring D, Lindon C, Phillips C, Itzhaki LS (2025b). Development of D-box peptides to inhibit the Anaphase Promoting Complex/Cyclosome. eLife.

[bib4] Greil C, Engelhardt M, Wäsch R (2022). The role of the APC/C and its coactivators Cdh1 and Cdc20 in cancer development and therapy. Frontiers in Genetics.

[bib5] Höfler A, Yu J, Yang J, Zhang Z, Chang L, McLaughlin SH, Grime GW, Garman EF, Boland A, Barford D (2024). Cryo-EM structures of apo-APC/C and APC/C^CDH1:EMI1^ complexes provide insights into APC/C regulation. Nature Communications.

[bib6] Isaacson RL, Díaz-Moreno I (2018). Editorial: Weak interactions in molecular machinery. Frontiers in Molecular Biosciences.

[bib7] Koszła O, Sołek P (2024). Misfolding and aggregation in neurodegenerative diseases: protein quality control machinery as potential therapeutic clearance pathways. Cell Communication and Signaling.

[bib8] Li K, Crews CM (2022). PROTACs: past, present and future. Chemical Society Reviews.

[bib9] Meng EC, Goddard TD, Pettersen EF, Couch GS, Pearson ZJ, Morris JH, Ferrin TE (2023). UCSF ChimeraX: tools for structure building and analysis.

[bib10] Müller L, Hoppe T (2024). UPS-dependent strategies of protein quality control degradation. Trends in Biochemical Sciences.

[bib11] Okoye CN, Rowling PJE, Itzhaki LS, Lindon C (2022). Counting degrons: lessons from multivalent substrates for targeted protein degradation. Frontiers in Physiology.

[bib12] Rodríguez-Gimeno A, Galdeano C (2025). Drug discovery approaches to target E3 ligases. Chembiochem.

[bib13] Thompson MA, De-Souza EA (2023). A year at the forefront of proteostasis and aging. Biology Open.

